# Inflammatory mediators-induced DNA damage in liver and brain injury: Therapeutic approach of 5-Methoy-N-acetyltryptamine

**DOI:** 10.1016/j.toxrep.2024.101816

**Published:** 2024-11-15

**Authors:** Mai O. Kadry, Hanaa Mahmoud Ali

**Affiliations:** a Therapeutic Chemistry Department, National Research Centre, El Buhouth St., Dokki 12622, Egypt; b Department of Genetics and Cytology, National Research Centre, Dokki 12622, Egypt

**Keywords:** Quercetin, DNA-damage, IL-6, CRP

## Abstract

The pathophysiology of hepatic and cerebral damage includes various molecular and signalling pathways. Assessment of inflammation-induced DNA damage is one of the principal issues for investigating organ distortion and mutation. The current study was premeditated to discover the prophylactic role of 5-Methoy-N-acetyltryptamine (5-MNAT) and/or Quercetin (QR) versus sodium nitrite (NaNO_2_) induced liver and brain injury in a rat model, as well as to clarify the different cross-linked inflammatory pathways and neurotransmitters. Pre-treatment with QR and 5-MNAT was followed with NaNO_2_ administration in a dose of (75 mg/kg BW). NaNO_2_-intoxication significantly caused alleviation in inflammatory biomarkers including C-reactive protein (CRP) and interleukin-6 (IL-6). The above-mentioned antioxidants noticeably amended the altered inflammatory biomarkers signalling pathways and liver function biomarkers including alanine aminotransferase (ALT) and lactate dehydrogenase (LDH). Furthermore, they modulated brain neurotransmitters including, GABA and serotonin in brain tissue. Likewise, the COMET assay revealed that these antioxidants successfully modified NaNO_2_-induced liver and brain DNA damage. In conclusion, treatment with both QR and 5-MNAT revealed the most effective therapeutic index in improving NaNO_2_-induced liver and brain injury, confirming the effectiveness of this combination as a powerful treatment for brain hypoxia. Nevertheless, this was confirmed with histopathological investigation.

## Highlights

• Induction of liver and brain injury via sodium nitrite.
• Treatment with 5-MNAT, Quercetin and their combination.
• Monitoring inflammatory mediators and DNA induced liver and brain damage.
• Monitoring brain neurotransmitters as GABA and serotonin.
• The combination regimen showed the most therapeutic impact.

## 1 Introduction

The release of numerous inflammatory mediators from glia and neuronal cells aggravates the impact of sodium nitrite-induced hypoxia on neuronal and hepatic tissue. IL-6, TNF-α, and IL-1β cytokines are known to be released during the early stages of hypoxia, causing either local or systemic inflammation or cell death. The vast majority of inflammation-triggered DNA damage is attributed to reactive nitrogen species (RNOS) which are released via immune cells to destroy pathogens or to counteract stress, on the other hand; they can also damage normal human cells. RNOS-induced DNA damage includes strand breaks in the phospho-diester backbone as well as nucleobase deamination, oxidation, and alkylation. DNA de-aminated products are mutagenic as they alter the site of hydrogen bonding leading to base mis-pairing [1]. Oxygen homeostasis is a key factor in the pathogenesis of different brain syndromes, and its disturbance plays an important role in various physiological progressions [2]. Hypokinetic hypoxia is a pathogenic process that is a consequence of inadequate blood flow contributing to decreased tissue oxygenation. Sodium nitrite (NaNO_2_) is an inorganic salt applied in meat treatment, colouring agents, and chemical manufacture. NaNO_2_ has a high affinity to haemoglobin (Hb) reducing its power to bind oxygen thus creating met-haemoglobinemia and producing hydrogen peroxide (H_2_O_2_) and nitrogen dioxide (NO_2_) contributing to hypoxia and liver injury [3]. Whereas, met-haemoglobin is oxidized by hydrogen peroxide to ferryl-Hb radical, and nitrogen dioxide oxidizes ferrous-Hb to met-haemoglobin. Reactive nitrogen species result in nitrosative stress this is well-thought-out as a significant mediator of cell structure damage, counteracting membranes, lipids, proteins and DNA. Hypoxia reduces brain antioxidant activity and raises lipid peroxidation levels [3]. Oxygen is very important for electrochemical impulse transfer among brain cells and for retaining neuronal function. Brain hypoxia, causes cell death within a few minutes this result in brain impairments and free radicals production [4]. The brain is very sensitive to stress-related degenerative stressors such as reactive oxygen species, including hydrogen peroxide, superoxide anion radical, and hydroxyl radical. These stressors cause brain membranes’ lipid peroxidation. Hypoxia influences lipids, proteins, carbohydrates, and electrolyte metabolism [5], [6]. According to Zedan, [7], NaNO_2_ administration consequences on oxidative stress, ischemia, inflammation, hypoxia and reduced metabolic energy, resulted in brain and liver injury [7]. Hypoxia damages hepatic, respiratory, digestive, nervous, urinary and endocrine systems [8]. Pro-inflammatory cytokines including interleukin 6 (IL-6) and tumour necrosis factor-α (TNF-*α*) play an essential role in inflammation and enhance the production of C-reactive protein (CRP) [9], [10]. Serotonin (monoamine neurotransmitter), excites respiratory rhythm neuromodulator [11], [12]. Disturbance in metabolites of serotonin in the cerebrospinal fluid in Prader-Willi disorder is linked with decreased ventilator response to hypoxia [13].

γ-amino butyric acid (GABA) originated via L-glutamate decarboxylation and exerts a dynamic role in the activity of sympathetic and endocrine systems and the control of blood pressure [14]. Hypoxia causes excessive production of glutamate, leading to seizure and neuronal damage in rat’s brain [15], [16].

N-acetyl-5-methoxytryptamine is an indole-derivative that crosses the blood-brain barrier and accumulates in the brain. It possesses ROS scavenger power and an anti-inflammatory role via NF-κB activation and transcription factors inhibition [17], NF-κB transcripts pro-inflammatory cytokines such as CRP, IL-6, and TNF-α and preserves DNA damage [18]. Quercetin is extracted from numerous fruits, beverages, and vegetables; it possesses anti-oxidative, anti-proliferative, and anti-inflammatory properties [18]. QR protects the lungs and brain in several injured models [19], [20], [21].

The current study was premeditated to discover the prophylactic role of 5-MNAT and/or QR versus NaNO_2-_induced liver and brain injury in a rat model, as well as to clarify the different cross-linked inflammatory pathways and neurotransmitters. The present research emphasizes the prophylactic impact of 5-MNAT and/or QR against brain injury caused by NaNO_2_. The antioxidants' potential against brain damage was assessed biochemically by measuring inflammatory biomarkers, neurotransmitter levels, and DNA fragmentation.

## 2 Materials and methods

### 2.1 Chemicals

Quercetin Cat #117–39–5 and Melatonin Cat # 73–31–4 were products of Sigma-Aldrich Co (St. Louis, MO, USA). Kits utilized for ALT and LDH determination were obtained from Randox Company (UK). All other chemicals were of the highest analytical grade.

### 2.2 Experimental animals

Forty (6 weeks old) Wistar male albino rats (120–150 g) were received from the National Research Centre Animal House, Egypt. Protocols outlined by the Experimental Animal Ethics Committee were followed. The animal Experimental protocol was approved by the Experimental Animal Ethics Committee of the National Research Centre (34512012023) and USA. Animals were retained under standard temperature and humidity conditions. They were allowed standard rat pellet chow with free access to tap water ad libitum.

Animals were divided into five groups of 8 rats each:

G 1: The control group received normal saline.

G 2: NaNO_2_- intoxicated rats with a single subcutaneous dose of (75 mg/kg) [22].

G 3: NaNO_2_- intoxicated rats pre-treated with QR (250 mg/kg, I.P) 24 h and 1 h before NaNO_2_ injection [22].

G 4: NaNO_2_- intoxicated rats pre-treated with 5-MNAT (250 mg/kg, I.P) 24 h and 1 h before NaNO_2_ injection [22].

G 5: NaNO_2_- intoxicated rats treated intra-peritoneal with a combination of QR (250 mg/kg) and 5-MNAT (250 mg/kg).

NaNO_2_, MNAT, and QR were emulsified in one drop of carboxyl methyl cellulose and then dissolved in normal saline.

Blood sampling and liver and brain tissue preparation occurred one hour post-NaNO_2_ injection; Isoflurane (3.8 ±1.1 min) followed by CO2 was used for anesthetize. Rats were then decapitated and sacrificed. Blood was taken and separated into two parts; the first was used for ALT, LDH, GABA, and serotonin determination while the second portion was coagulated and centrifuged to prepare serum. Sera were maintained at −80 °C for biochemical analysis. To prepare 20 % homogenate, rats' liver, and brain tissues were separated and gently homogenized in phosphate buffer. A portion of the tissues were kept in formalin for histopathological investigation.

### 2.3 Biochemical serum investigation

Interleukin-6 was assessed by ELISA kit (IBL- International GmbH-Flughafenstr-Hamburg- Germany).

CRP was analysed by immunome phelometric (Dade Behring N Latex High Sensitivity- CRPTM mono-assay) on a Behring Nephelometer-II analyser [22].

### 2.4 ALT, LDH, and NOX determination

ALT, LDH, and NOX were estimated spectrophotometry via Randox Company (USA) assay kits according to manufacturer instructions [23].

### 2.5 Gamma-aminobutyric acid (GABA) and serotonin Assay

Quantitative assessment of concentrations of serotonin [24] and GABA [25] in tissues of the brain was achieved by ELISA kits, following the manufacturer’s procedure. The Rat ELISA kits were purchased from MyBiosource Co. serotonin (Cat # MBS725497) and GABA (Cat # MBS045103). Briefly, x phosphate-buffered saline (PBS, pH 7.0) was used to remove extra blood. For serotonin assay, the homogenate was made 1:1 w/v (homogenization of (500 mg) of tissue of the brain in (500 μL) PBS on ice) whereas homogenate of GABA was formed 1:10 (10 mg tissue in 100 μL PBS). Next, centrifugation (at 4000 rpm; 15 min) of the homogenates was performed. This protocol depends on a competitive enzyme immunoassay method utilizing polyclonal anti-SER / GABA antibodies, and GABAHRP serotonin conjugates. Blank is prepared by (100 μL) brain sample, BPS (as) or standard then incubated with 50 μL serotonin / GABAHRP conjugates (within a pre-coated plate) for 1 h at 37 °C. 100 μL of HRP enzyme substrate was poured into the wells after washing, and the plate was incubated (15 min) in the dark (until the production of the blue colour). Lastly, stop solution (50 μL) was added, this will cause the solution to turn yellow. The spectrophotometer is used to measure the intensity of colour (at 450 nm).

### 2.6 Comet assay

The comet assay (single-cell gel electrophoresis) is a straightforward method for quantifying strand breakage in deoxyribonucleic acid (DNA) in eukaryotic cells in liver and brain tissues. Nucleoids are formed when cells placed in agarose on a microscope slide are lysed with detergent and high salt to generate supercoiled loops of DNA linked to the nuclear matrix. Fluorescence microscopy reveals comet-like structures formed by high-pH electrophoresis; the intensity of the comet tail relative to the head represents the amount of DNA breaks. The most likely explanation is that loops with a break lose supercoiling and are free to stretch toward the anode [26].

### 2.7 Histopathological investigation

Liver and brain sections were fixed, processed, and embedded in paraffin, then were cut in about (5μm) thickness using a microtome. Finally, they were stained with H&E stain and investigated under an electron microscope [27].

### 2.8 Statistical analysis

Data were presented as means ± SEM. The statistics were accomplished using a one-way analysis of variance (ANOVA) followed by the Tukey-Kramer multiple comparisons test. The level of significance was set at p≤ 0.05. Statistical tests were conducted using SPSS 21 (IBM, USA). Graphs were performed with the graphpad prism 10 program.

## 3 Results

### 3.1 Impact of QR and/or 5-MNAT on inflammatory biomarkers

**Fig. 1:**
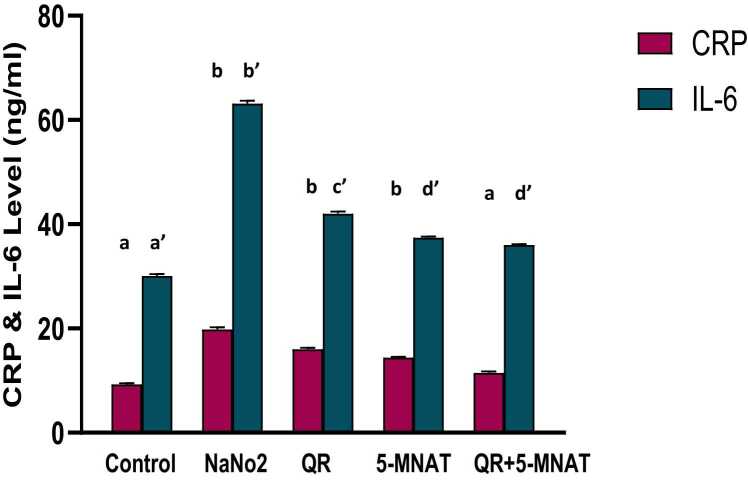
Impact of Quercetin (QR), 5-Methoy-N-acetyltryptamine (5-MNAT) and their combination on serum, C-reactive protein (CRP) and interleukin-6 (IL-6) levels following sodium nitrite (NaNO_2_) induced hypoxia. Data are expressed as means ± SEM (n=8). Different letters are significantly different from each other at P≤0.05.

The level of immunological pro-inflammatory cytokines, including CRP and IL- 6 in NaNO_2_-intoxicated rats was markedly elevated by a mean value of (20 & 60 ng/ml) respectively than that of control values. Pre-treatment with QR and/or 5-MNAT noticeably reduced the induced inflammatory mediators, with a mean value of (15 & 40 ng/ml) for QR and (13 & 35 ng/ml) for 5-MNAT respectively compared with NaNO_2_-intoxicated animals with the combination regimen showing the most significant impact at (P< 0.05) (Fig. 1).

### 3.2 Impact of QR and/or MNAT on ALT, LDH, and NOX activities

**Fig. 2:**
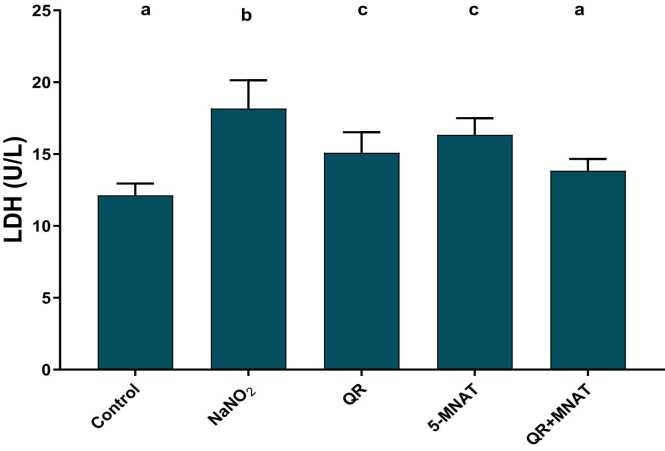
Impact of Quercetin (QR), 5-Methoy-N-acetyltryptamine (5-MNAT) and their combination on serum LDH level following sodium nitrite (NaNO_2_) induced hypoxia. Data are expressed as means ± SEM (n=8). Different letters are significantly different from each other at P≤0.05.

**Fig. 3:**
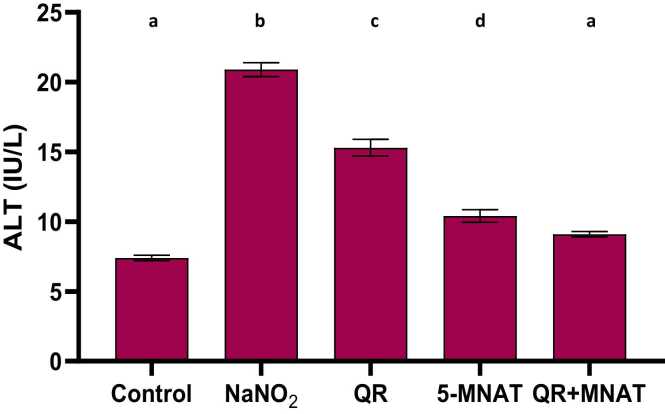
Impact of Quercetin (QR), 5-Methoy-N-acetyltryptamine (5-MNAT) and their combination on serum ALT level following sodium nitrite (NaNO_2_) induced hypoxia. Data are expressed as means ± SEM (n=8). Different letters are significantly different from each other at P≤0.05.

**Fig. 4:**
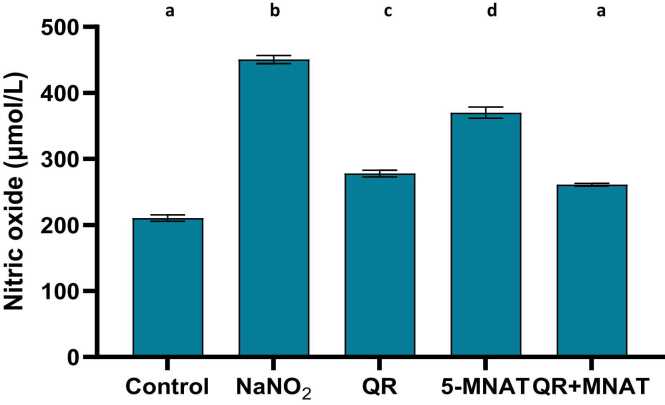
Impact of Quercetin (QR), 5-Methoy-N-acetyltryptamine (5-MNAT) and their combination on serum nitric oxide (NOX) level following sodium nitrite (NaNO_2_) induced hypoxia. Data are expressed as means ± SEM (n=8). Different letters are significantly different from each other at P≤0.05.

NaNO_2_-induced a significant elevation in ALT and LDH with a mean value of (22 & 18 U/L) in addition to, NOX levels with a mean value of (440 μmol/L) as compared with the control value (P < 0.05). QR, 5-MNAT, and their combination significantly restored ALT, LDH, and NOX levels near the normal value with a mean value of (15, 14 & 240) for QR and (10, 17 & 350) for 5-MNAT and (8, 13 &220) for the combination respectively with the combination regimen showing the most significant impact (P< 0.05), Fig. 2, Fig. 3, Fig. 4.

### 3.3 Impact of QR and/or MNAT on neurotransmitters

**Fig. 5:**
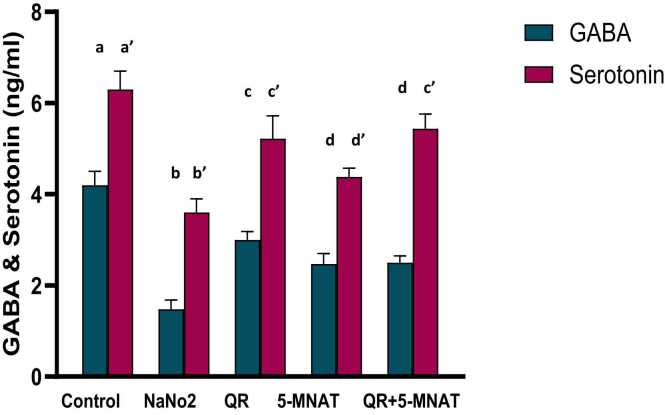
Impact of Quercetin (QR), 5-Methoy-N-acetyltryptamine (5-MNAT) and their combination on serum, GABA and serotonin levels following sodium nitrite (NaNO_2_) induced hypoxia. Data are expressed as means ± SEM (n=8). Different letters are significantly different from each other at P≤0.05.

It was obvious that NaNO_2_ induced a marked reduction in the neurotransmitters GABA and serotonin with a mean value of (1.5 & 3.2 ng/ml) respectively (Fig. 5). On the other hand pre-treatment with either QR or 5-MNAT solely improved the amelioration in serotonin, and GABA levels with a mean value of (2.5 & 5 ng/ml) for QR and (2.1 & 4.6 ng/ml) for 5-MNAT respectively as compared with the NaNO_2_ rats. Meanwhile, the combination of QR and 5-MNAT was the most effective with a mean value of (2.4 & 5.5 ng/ml) respectively.

### 3.4 Impact of QR and/or MNAT on liver and brain DNA damage

**Fig. 6:**
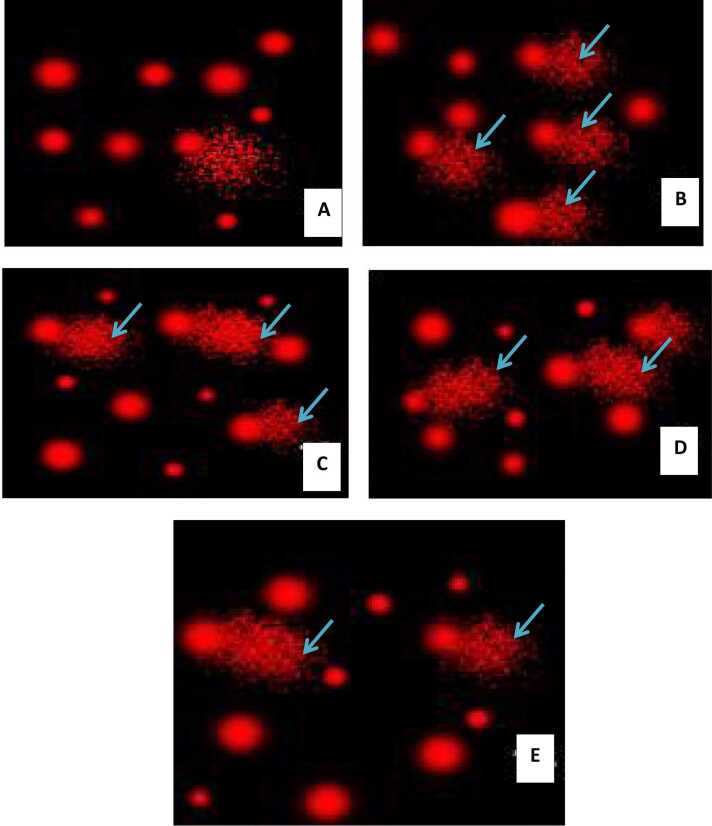
COMET assay showing the degree of DNA damage and tail length in liver tissues (A) Normal control group showing No DNA damage (B) NaNO_2_ intoxicated group revealing high percent of DNA damage (C) NaNO_2_ group treated with QR revealing moderate DNA damage (D) 5-MNAT treated group revealing moderate DNA damage (E) Group treated with both QR and 5-MNAT revealing low DNA damage.

**Fig. 7:**
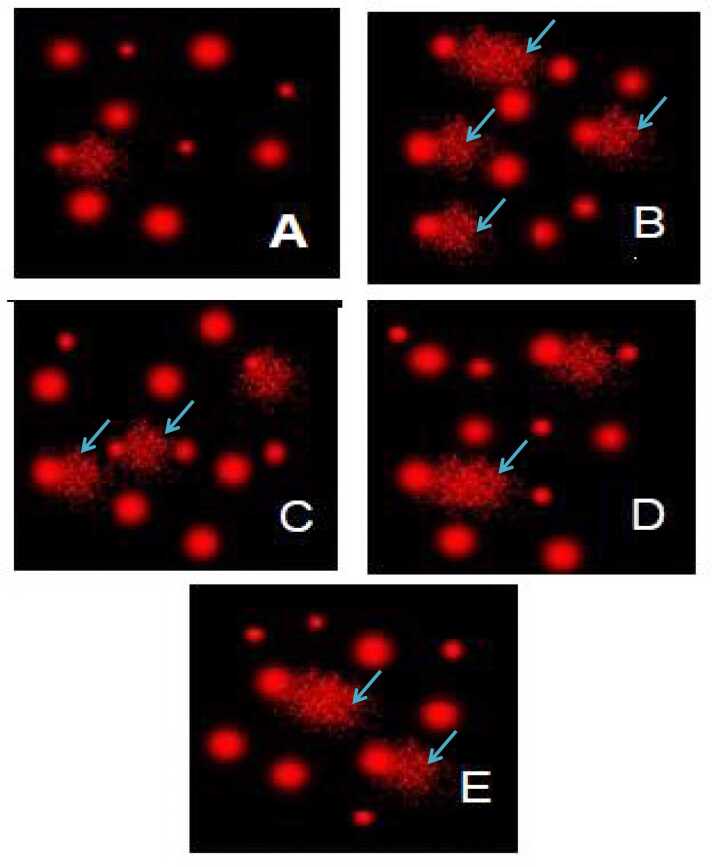
DNA damage in the brain tissues of NaNO_2_- intoxicated rats and the effect of QR or/and MN treatment on DNA damage level. COMET assay showing the degree of DNA damage in the brain tissues of (A) Normal control group (B) Group intoxicated with the NaNO_2_ declaring great DNA damage obvious with the tail length (C) QR treated group with moderate DNA damage (D) NaNO_2_ intoxicated group treated with 5-MNAT showing low DNA damage (E) Intoxicated group treated with QR and 5-MNAT with very low DNA damage.

A marked increase in the tail length, moment and DNA percentage (tail DNA content) with a mean value of (4 & 3; 14.26 & 10.15; 3.5 & 3.6) respectively was revealed in the NaNO_2_-intoxicated rats as compared with the control group reflecting the tremendous DNA damage in both brain and liver tissues. QR and/or 5-MNAT pre-treatment reduced DNA damage, as presented via depletion in the aforementioned indicators of DNA damage, compared with intoxicated rats. Fig. 6, Fig. 7 represent the modulatory impact of 5-MNAT and/or QR on liver and brain DNA damage post elevation in the NaNO_2_- groups. QR showed moderate DNA damage meanwhile, 5-MNAT and the combination regimen reflected low DNA damage with a mean value of (2.5 & 2.9 %) respectively. As the percentage of DNA damage increases the tail length increases due to the migration of damaged DNA to the tail (Table 1, Table 2).

**Table 1:** Impact of QR, 5-MNAT and the combination regimen on brain DNA damage.

Brain Groups	% Tailed	% Untailed	μm tail length	Tail DNA %	Unit Tail Moment
Control	4	96	1.21±0.01^**a**^	1.53	1.85±0.11^**a**^
NaNO_2_	12	88	4.05±0.02^**b**^	3.52	14.26±0.65^**b**^
QR	10	90	2.55±0.12^**a**^	2.55	6.50±0.21^**c**^
5-MNAT	7	93	2.55±0.29^**a**^	3.26	8.31±0.43^**c**^
QR+MNAT	6	94	3.33±0.24^**c**^	3.05	10.16±0.38^**d**^

Data are expressed as mean ± SEM. Different letters are significantly different from each other while similar letters aren’t significantly different from each other at P≤0.05.

**Table 2:** Impact of QR, 5-MNAT and the combination regimen on liver DNA damage.

Liver Groups	% Tailed	% Untailed	μm tail length	Tail DNA %	Unit Tail Moment
Control	3	97	2.54±0.32^**a**^	2.11	5.36 ±0.4^**a**^
NaNO_2_	11	89	2.84±0.25^**a**^	3.57	10.15 ±0.8^**b**^
QR	7	93	2.24±0.19^**a**^	2.90	6.50 ±0.3^**a**^
5-MNAT	8	92	3.56±0.17^**b**^	3.22	11.48 ±0.5^**b**^
QR+MNAT	5	95	3.07±0.28^**b**^	2.95	9.06 ±0.6^**b**^

Data are expressed as mean ± SEM.Different letters are significantly different from each other while similar letters aren’t significantly different from each other at P≤0.05.

### 3.5 Histopathological investigation

**Fig. 8:**
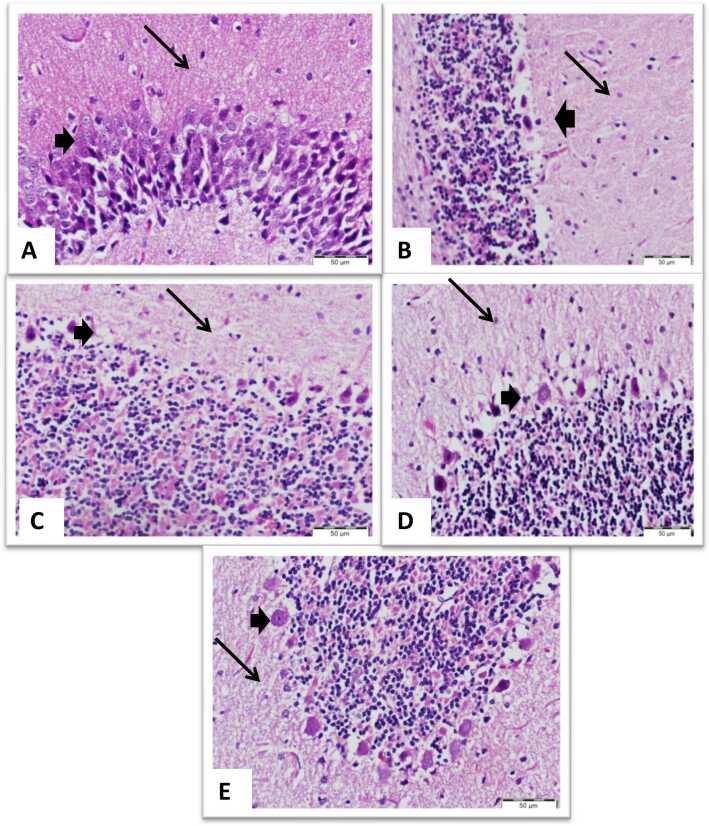
Brain sections (Scale bar: 50 µm) (A) Normal control neuronal cells (arrow head) and nerve fibers and neuroglia cells (arrow) (B) NaNO_2_ intoxicated rats showing marked degeneration of neuronal cells (arrow head) while neutrophil does not change (arrow) (C, D and E) Brain of rats received 5-MNAT, Quercetin and combination treatments respectively, showing normal neurons (arrow heads) specially those received combination of both 5-MNAT and Quercetin regimen (E).

**Fig. 9:**
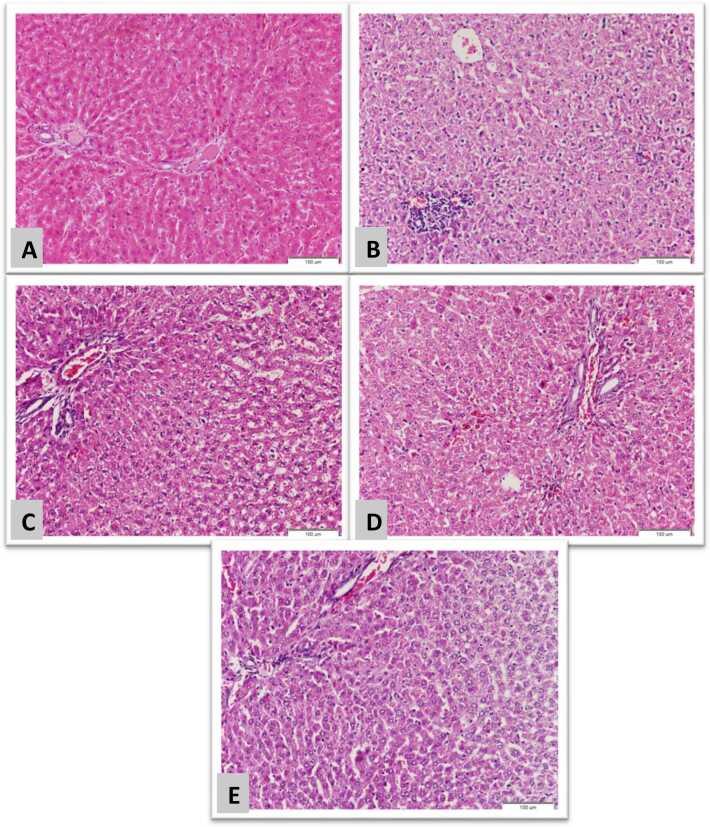
Liver sections stained with H&E (Scale bar: 100 µm) (A), Normal control hepatocytes and portal area. (B) NaNO_2_ group showing marked hepatocellular degeneration, both cytoplasmic and nuclear in a form of pyknosis. Also there are foci of inflammatory cellular infiltration. (C, D and E) Groups received 5-MNAT, Quercetin and combination treatment respectively, showing marked improvement in the cellular degeneration and a marked decrease in the cellular infiltration specially in rats received combination of both melatonin and Quercetin regimen.

Fig. 8, Fig. 9 represent sections of the brain exposed to NaNO_2_-induced hypoxia showing marked degeneration of neuronal cells while neutrophil does not change meanwhile, sections of the brain from a rat exposed to hypoxia and received 5-MNAT, Quercetin, and a combination of both respectively, showing normal neurons specially those received combination of both 5-MNAT and Quercetin. On the other hand, a section of the liver from rats exposed to hypoxia showed marked hepatocellular degeneration, both cytoplasmic and nuclear in the form of pyknosis. Also, there are foci of inflammatory cellular infiltration. While, those who received 5-MNAT, Quercetin, and a combination of both, showed marked improvement in cellular degeneration and a marked decrease in cellular infiltration especially in rats who received a combination regimen.

## 4 Discussion

Hypoxia interrupts all body metabolic reactions, causing insufficient brain energy supply leading to death and vital organs damage [28]. Nitrites cause toxicity via met-haemoglobinmia which is characterized by hypoxic tissues leading to death and hepatic influence [29].

In this study, the anti-hypoxic impact of QR and 5-MNAT against NaNO_2_-induced liver and brain injury in the NaNO_2_-hypoxic rat model was evaluated via inflammatory and DNA damage biomarkers.

NaNO_2_ elucidated an inflammatory response confirmed via the release of inflammatory mediators, such as CRP and IL-6. TNF-α plays a vigorous role in inflammation and is formed via activated macrophages. NaNO_2_ -administration destroy cells, DNA, and cell membranes resulting in cell necrosis and mutation [30]. Previous researches revealed elevated levels of TNF*-α* and IL-6 in rats exposed to hypoxic environments. Hypoxia activates NF-*k*B and triggers TNF*-α* and IL-6 release, which are correlated to brain and liver DNA damage via reactive nitrogen species production [31]. On the other hand, pre-administration of the examined antioxidants, preceding NaNO_2_ induction significantly reduced NaNO_2_-induced inflammatory biomarkers including CRP and IL-6. The advantageous impact of QR and 5-MNAT is highly related to their immune-modulatory and anti-inflammatory power. QR or 5-MNAT may protect from the inflammation that is enhanced by different pathological circumstances via the decline in these intermediaries [32], [33]. 5-MNAT reduces inflammation by attenuating the NF-*k*B and Nrf2 cascades and prevents inflammatory mediators TNF-α and IL-6 in hypoxic animals [34]. QR elucidates its anti-oxidant power via RNS and ROS scavenging. It increases endothelial NO and improves vasodilatation via relaxing smooth muscle thus enhancing vasodilation and angiogenesis in response to hypoxia [33]. QR hinders ischemia-reperfusion damage via apoptosis inhibition and inhibits the mitochondria-dependent caspase cascade [33].

NaNO_2_ revealed a significant elevation in ALT, LDH, and NOX levels in NaNO_2_-intoxicated rats as compared with the control value. Conversely, QR, 5-MNAT, and their combination significantly returned ALT, LDH, and NOX. This coincides with that NaNO_2_-binding to oxy-Hb disrupts the bounded O_2_ and forms meth-Hb, hydrogen peroxide, and nitric dioxide the initiating step for free radical chain reaction [35]. Likewise, hydrogen peroxide oxidizes met-Hb into ferryl- haemoglobin radical; however, nitric dioxide oxidizes ferrous haemoglobin into met-haemoglobin. Accordingly, this hinders O_2_-binding to haemoglobin, contributing to the fall in blood capacity to bind O_2_, encouraging hypoxia. Hypoxia triggers liver oxidative stress via lipid peroxidation thus causing leakage of liver enzymes from the liver tissues [36]. The protective efficiency of QR depends on its anti-inflammatory and anti-oxidant impact. QR impairs Nrf2 nuclear translocation, it controls the anti-oxidant reaction and ROS rise by preventing the activities of xanthine oxidase and NADPH oxidase. Oxidative stress and inflammation are likewise linked to NaNO_2_ toxic effects [37], [38].

Neurotransmitters elicit a vital role in controlling brain normal physiological systems. O_2_-requiring rate-limiting enzymes control neurotransmitters’ synthesis. Thus, hypoxia may influence neuronal functions via neurotransmitter modification [39].

Brain catecholamine and serotonin levels decline in neurodegenerative disorders. Coincide with previous research, the hypoxia-enhanced brain injury model triggered a decline in serotonin and GABA levels, this may be correlated to the alleviated monoamine oxidase-A activity, which is activated via NaNO_2_ initiating the degradation and the reduction in brain monoamines. Likewise, NaNO_2_-induced hypoxia diminishes tryptophan hydroxylase and tyrosine hydroxylase activity, which are vital enzymes that play a vital role in the synthesis of serotonin and catecholamine [40].

Hypoxia elucidates an extreme generation of glutamate contributing to neuronal damage. γ-amino-butyric acid exerts neuro-protection and antagonizes brain toxicity [41]. Consequently, the observed depletion in GABA levels in the existing study may be correlated with brain impairment in hypoxic rats. The declined GABA in this hypoxic model harmonizes with that GABA was significantly declined in hypoxic-neonatal rats' brain stem and cerebellum via inhibiting the GABA-synthetic pathway and reducing the expression of glutamate decarboxylase. Growing evidence observed GABA receptors (GABA_A_-Rs) on most immune cells that trigger anti-inflammatory reactions of T cells. Contrary, GABA_A_-Rs antagonist triggers pro-inflammatory action of T cells and promotes the release of CD8+ and CD4+ that enhance the production of inflammatory mediators IL-6, IFNγ and IL-17 on the other hand, inhibit anti-inflammatory mediator IL-10 [42]. Pre-treatment with QR, 5-MNAT and their combination significantly improved the neurotransmitters' reduced levels. Melatonin revealed mitochondria protective powers, contributing to the rise in calcium influx, ATP production and reduction in oxidative stress. 5-MNAT influences striatal dopamine levels via the enhancement of monoamine synthesis [42]. QR improved the affected neurotransmitters’ level (serotonin, dopamine, noradrenaline, GABA) in the striatum. The rise in neurotransmitters’ level is correlated to QR inhibitory activity or owed to the reduction in neural oxidative stress [43], [44], [45], [46].

Inflammation-triggered DNA damage is contributed to RNOS released via immune cells to counteract pathogens. RNOS-induced DNA damage includes phosphor-diester backbone strand breaks as well as DNA-base deamination, oxidation and alkylation [1]. DNA de-aminated products are mutagenic as they alter the site of hydrogen bonding leading to base mis-pairing. Sodium nitrite intoxication significantly triggered DNA damage. This may be enlightened by the reaction of NO with O_2_.- to create a vigorously reactive ONOO^−^ (per-oxynitrite) radical. Nitrotyrosine is generated via ONOO^−^ conjugate with proteins and is a nitrosative stress-marked indicator. Nitrotyrosine conjugates with DNA, phospholipids, and proteins contributing to the formation of products responsible for pathogenesis and exhibiting numerous mutagenic, genotoxic, and cytotoxic impacts such as DNA and protein synthesis inhibition and enzyme inactivation [47]. NaNO_2_-intermediated DNA damage is possible because of several mechanisms: free radicals initiating lipid peroxidation species or free radicals triggering chemical alterations [47].

On the contrary, pre-administration of QR and/or 5-MNAT efficiently alleviated brain DNA damage. The preceding results are in harmony with that Quercetin possesses anti-inflammatory, antiviral, and antitumor impact [48], [49], [50] and Melatonin improves brain stress biomarkers that contribute to DNA damage. Melatonin free radical scavenger power likewise, presents a defensive mechanism contrary to DNA damage [51], [52].

## 5 Conclusion

It is concluded that QR and 5-MNAT combination possesses a synergistic prophylactic power against inflammation, DNA damage, and the depletion in neurotransmitters enhanced through hypoxia in the brain and liver tissues. In conclusion, treatment with QR and 5-MNAT was effective; their combination exerts neuro-protection and hepato-protection in hypoxic rats by numerous mechanisms depending on their anti-oxidant synergistic power. This machinery includes mitigating DNA damage, and anti-inflammation, along with the depletion of the decreased biogenic amines and GABA levels.

## Study limitation

The number of the used animals and measured parameters caused some limitations to the experiment. Sodium nitrite handling may cause asthma attacks with breath shortness, cough, wheezing, and chest tightness. It may affect the thyroid, heart, kidneys, and liver. Repeated exposure can cause lung fibrosis even if no signs are observed. Animal experiments don't exactly mimic the way that the human body and diseases may respond to treatments, chemicals, or drugs. Some compounds are highly toxic so small amounts may cause death. Future studies should investigate the Sodium nitrite effect on different organs such as the brain, testis, and lung.

## Funding

This study has no financial support.

## CRediT authorship contribution statement

**Hanaa Mahmoud Ali:** Writing – review & editing, Writing – original draft, Visualization, Validation, Supervision, Resources, Methodology, Investigation, Funding acquisition, Formal analysis, Data curation, Conceptualization. **Mai O. Kadry:** Writing – review & editing, Writing – original draft, Visualization, Validation, Supervision, Software, Resources, Project administration, Methodology, Investigation, Formal analysis, Data curation, Conceptualization.

## Declaration of Competing Interest

The authors declare that they have no known competing financial interests or personal relationships that could have appeared to influence the work reported in this paper.

## Data Availability

No data was used for the research described in the article.
